# Human papillomavirus vaccination of girls in the German model region Saarland: Insurance data-based analysis and identification of starting points for improving vaccination rates

**DOI:** 10.1371/journal.pone.0273332

**Published:** 2022-09-02

**Authors:** Anna Sternjakob-Marthaler, Barbara Berkó-Göttel, Jürgen Rissland, Jakob Schöpe, Emeline Taurian, Hanna Müller, Gero Weber, Stefan Lohse, Thomas Lamberty, Bernd Holleczek, Harry Stoffel, Gunter Hauptmann, Martin Giesen, Christiane Firk, Alexandra Schanzenbach, Florian Brandt, Heike Hohmann, Quirin Werthner, Dominik Selzer, Thorsten Lehr, Stefan Wagenpfeil, Sigrun Smola

**Affiliations:** 1 Institute of Virology, Saarland University Medical Center, Homburg, Germany; 2 Helmholtz Institute for Pharmaceutical Research Saarland (HIPS), Helmholtz Centre for Infection Research, Saarbrücken, Germany; 3 Institute for Medical Biometry, Epidemiology and Medical Informatics, Saarland University Medical Center, Homburg, Germany; 4 Physical Geography and Environmental Research, Saarland University, Saarbrücken, Germany; 5 Ministry of Health, Social Affairs, Women and the Family, Saarbrücken, Germany; 6 Saarland Cancer Registry, Saarbrücken, Germany; 7 Kassenärztliche Vereinigung Saarland, Saarbrücken, Germany; 8 AOK Rheinland-Pfalz/Saarland, Saarbrücken, Germany; 9 IKK Südwest, Saarbrücken, Germany; 10 Clinical Pharmacy, Saarland University, Saarbrücken, Germany; The University of the South Pacific, FIJI

## Abstract

In Germany, the incidence of cervical cancer, a disease caused by human papillomaviruses (HPV), is higher than in neighboring European countries. HPV vaccination has been recommended for girls since 2007. However, it continues to be significantly less well received than other childhood vaccines, so its potential for cancer prevention is not fully realized. To find new starting points for improving vaccination rates, we analyzed pseudonymized routine billing data from statutory health insurers in the PRÄZIS study (prevention of cervical carcinoma and its precursors in women in Saarland) in the federal state Saarland serving as a model region. We show that lowering the HPV vaccination age to 9 years led to more completed HPV vaccinations already in 2015. Since then, HPV vaccination rates and the proportion of 9- to 11-year-old girls among HPV-vaccinated females have steadily increased. However, HPV vaccination rates among 15-year-old girls in Saarland remained well below 50% in 2019. Pediatricians vaccinated the most girls overall, with a particularly high proportion at the recommended vaccination age of 9–14 years, while gynecologists provided more HPV catch-up vaccinations among 15-17-year-old girls, and general practitioners compensated for HPV vaccination in Saarland communities with fewer pediatricians or gynecologists. We also provide evidence for a significant association between attendance at the children´s medical check-ups “U11” or “J1” and HPV vaccination. In particular, participation in HPV vaccination is high on the day of U11. However, obstacles are that U11 is currently not financed by all statutory health insurers and there is a lack of invitation procedures for both U11 and J1, resulting in significantly lower participation rates than for the earlier U8 or U9 screenings, which are conducted exclusively with invitations and reminders. Based on our data, we propose to restructure U11 and J1 screening in Germany, with mandatory funding for U11 and organized invitations for HPV vaccination at U11 or J1 for both boys and girls.

## Introduction

Invasive cervical cancer is a consequence of persistent human high-risk papillomavirus (HPV) infection [1, 2]. Cervical cancer remains the most important vaccine-preventable HPV-associated cancer, with 604,127 cases and 341,831 deaths in 2020 worldwide [3]. In Germany, the incidence of cervical cancer in 2016 was 8.7 per 100,000 women (age-standardized, European population [4]), while neighboring countries such as Switzerland, Austria, France and the Netherlands have lower incidences [4, 5]. For cancers of the vagina, vulva, penis, anus, and oropharynx, HPV-associated and HPV-independent forms of cancer are existing [6, 7]. In recent years, there has been a particularly sharp increase in potentially HPV-associated oropharyngeal cancers in women in Germany [8, 9]. The same is true for vulvar cancer [7, 10]. In particular, the incidence of vulvar cancer in Germany is also relatively high compared to other European countries [4]. With the exception of cervical cancer in women and human immunodeficiency virus (HIV)-positive men who have sex with men, who have a 100-fold increased risk of anal cancer, there is currently no organized screening in Germany for HPV-associated cancers [11–14].

HPV vaccination, with high immunogenicity particularly in preadolescents, high efficacy, and a good safety profile, is an effective measure for sustained reduction in the burden of HPV-associated cancers. Because HPV is sexually transmitted, vaccination is most efficacious when given prophylactically to HPV-naïve individuals, ideally before first sexual intercourse. Population studies from other countries with school-based vaccination such as Australia show high vaccination rates (>80%) providing herd immunity in the population beyond individual protection. This makes cervical cancer elimination a realistic goal [15]. In Australia, the 4-valent and subsequent 9-valent vaccines have been used, covering the high-risk viruses HPV16, 18 (4-valent) or 16, 18, 31, 33, 45, 52, and 58 (9-valent), as well as the low-risk viruses HPV6 and 11. Already in 2013 a substantial reduction in cervical precancerous lesions was observed [4]. A meta-analysis found that in countries with HPV vaccination rates >50% in girls, there was a 68% reduction in HPV16 and 18 infections as early as 2014 [16]. More recent meta-analyses of studies between 2014 to 2018 showed a decrease in HPV16 and 18 infections by 83% 5–8 years after vaccination introduction in 13- and 19-year-old females, and of anogenital warts by 67% in 15-19-year-old females [17]. Laryngeal papillomas also showed a significant decrease over the course of HPV vaccination [18]. Herd immunity was reflected in an 81% and 51% reduction in genital warts in heterosexual men under 21 and 21–30 years of age, respectively [19]. A reduction of high-grade cervical dysplasia (CIN2+) by 51% in young women aged 15–19 years and by 31% in 20–24 year-old females was observed [17]. Most importantly, a Swedish registry study of females aged 10–30 years conducted between 2006 and 2017 found a substantially lower risk of invasive cervical cancer at the population level (88% reduction after covariate adjustment) if HPV vaccination was administered before the age of 17 years [20]. This indicates that timely HPV vaccination is not only able to prevent viral infection, benign warts, and precancerous lesions but also cancer.

In Germany, the HPV vaccination for girls has been recommended by the Standing Committee on Vaccination (STIKO) in 2007 and has since been one of the mandatory benefits of statutory health insurance. Originally, the vaccination age was set at 12–17 years [21]. In 2014, the vaccination age was lowered to 9–14 years and catch-up vaccination was recommended until the age of 17 years [22]. In addition, the vaccination schedule was adjusted to the age of the vaccinated. For 9-14-year-old children, 2 doses are recommended, with the second dose given 5–13 month after the first. Three doses are given, if the interval between doses 1 and 2 is less than 5 month or if the child is older than 15 years (S1 Table). In 2018, the HPV vaccination recommendation was extended to boys [23], as about 6,250 women and about 1,600 men developed HPV-related carcinomas of the cervix, vagina, vulva, anus, penis, or oropharynx every year in Germany [24]. HPV vaccination in Germany is recommended but still opportunistic, as there is not yet a comprehensive school-based or otherwise organized HPV vaccination program. The Robert Koch-Institute (RKI) monitors HPV vaccination rates in Germany. According to the RKI, only 31.3% of 15-year-old girls were fully vaccinated in 2015 [25], and HPV vaccination was far less well received than other childhood vaccines. Thus, the potential of cancer prevention through vaccination is not fully realized, and there is an urgent need to find entry points for improving HPV vaccination rates.

The aim of the study PRÄZIS (prevention of cervical carcinoma and its precursors in women in Saarland) was to analyze factors influencing HPV vaccination in Saarland on the basis of routine health insurance billing data for HPV vaccination and other health measures, such as preventive examinations for children, and thus to identify starting points for improving the uptake of HPV vaccination.

We selected Saarland as a model region, a federal state in western Germany with relatively low migration [26] and relatively high incidence rates for cervical, vulvar, and head and neck cancers in women [4]. Data were provided by the Association of Statutory Health Insurance Physicians Saarland (Kassenärztliche Vereinigung Saarland, KVS) and two statutory health insurance funds, AOK Rheinland-Pfalz/Saarland (AOK) and IKK Südwest (IKK). In Saarland preventive check-ups for children to the age of 5 years (up to the “U9”-examination) are mandatory and centrally organized. Screenings at older ages, on the other hand, are voluntary. Two examinations exist at the HPV vaccination age, the “U11” (age 9–10) and “J1” (age 12–14). While the U11 is currently not offered by all statutory health insurers, the “J1” visit is financed and offered but not mandatory (S2 Table).

Our study provided new findings, particularly on the age-specific implementation of HPV vaccination by specific medical specialist groups and on the uptake of HPV vaccination compared with other preventive measures among girls and young women. We found that pediatric medical check-up examinations at the HPV vaccination age of 9–14 years, the “U11” and “J1” examinations, can provide an excellent opportunity to deliver HPV vaccination in an age-appropriate manner. Specifically, our data indicate that there is a significant association between participation in U11 or J1 check-ups and HPV vaccination, and that the day of U11 has a high uptake for HPV vaccination in the year of the U11 preventive examination.

## Material and methods

### Ethical statement

In this study, we analyzed pseudonymized prospective and retrospective statutory health insurance billing data. The Ethics Committee of the Saarland Medical Association reviewed, approved all study procedures, and waived the requirement for informed consent (ethical vote Nr. 186/17). All procedures were performed in accordance with the recommendations of the data protection authorities (Saarland University, Saarland Ministry of Social Affairs, Health, Women and Family Affairs, Saarland Independent Data Protection Center).

### Data sources

Pseudonymized routine data available from the KVS (2013–2019), IKK (2012–2019) and AOK (2009–2018) served as data sources. In addition to general information, service codes according to the uniform valuation standard (EBM) represented an essential component of the analyzed variables. Where necessary, the data from KVS and IKK were linked by automated record linkage in a study database and analyzed. The U11 check-up is not offered or not billed via the KVS by all statutory health insurance funds in Saarland. For our study, data on the U11 examination were only available from IKK. The procedure of pseudonymization is described in S1 File.

### Inclusion criteria and data sets

The KVS and IKK data sets contained data on females who had received services from physicians in Saarland (S3 Table). This included individuals who resided outside of Saarland (S1 Fig). Saarland residents were selected by zip code. Missing data on gender were imputed if the gender could be inferred from more than 50% of the remaining data. The data sets and study populations used to answer the respective questions are listed in the corresponding figure and table legends.

An overview of the data sets used and the operationalization of the variables for the individual questions is provided in Table 1. Numbers of girls included in the analyses for the different research questions are shown in the respective supporting tables (S4–S8 Tables).

**Table 1:** Overview of the data sets used and the operationalization of the variables for the individual research questions.

Research question	Study population	Data basis	Operationalization of the variables
Age at HPV vaccination date	females in Saarland, birth year 1996–2010	KVS	zip code,
		(2013–2019)	birth date (dd/mm/jjjj),
			date of HPV vaccination (dd/mm/jjjj),
			EBM: 89110A, 89110B
Complete HPV vaccination	females in Saarland, birth year 2000–2004	KVS	zip code,
		(2013–2019)	birth date (dd/mm/jjjj),
			date of HPV vaccination (dd/mm/jjjj),
			EBM: 89110A, 89110B
HPV vaccination in time	females in Saarland	KVS	zip code,
		(2013–2019)	birth date (dd/mm/jjjj),
		AOK	EBM: 89110A, 89110B, 01820, 01821, 01822, 01825, 32132
		(2009–2018)	
Average number of doctor visits at the HPV vaccination age	females in Saarland, birth year 2002–2010	KVS	zip code,
		(2013–2019)	birth date (dd/mm/jjjj),
			date of first HPV vaccination (dd/mm/jjjj),
			EBM: 89110A, 89110B;
			Average number of doctor visits (2013–2019)
Effect of the place of residence of the vaccinee	females in Saarland	KVS (2013–2019)	zip code,
			birth date (dd/mm/jjjj),
			date of HPV vaccination (dd/mm/jjjj),
			EBM: 89110A, 89110B
HPV vaccinating physician specialist group	females in Saarland, birth year 1996–2010,	KVS	*Patient*: zip code,
		(2013–2019)	birth date (dd/mm/jjjj),
			date of HPV vaccination (dd/mm/jjjj),
	contract physicians in Saarland		
			EBM: 89110A, 89110B,
			physician-ID (LANR)
			*physician*:
			zip code, physician specialist group in Saarland,
			physician-ID (LANR),
			Number of HPV vaccinations given per year
			(2013–2019),
			Number of other vaccinations given per year
			(2013–2019), EBM:
			89100R, 89116R, 89201R, 89302R, 89303R, 89400R
HPV vaccination date compared to U11 visit	females in Saarland, birth year 2003–2010	IKK	zip code,
		(2013–2019) from linked KVS-dataset	birth date (dd/mm/jjjj),
			date U11 (dd/mm/jjjj),
	participation rate birth year 2004–2007		EBM: 81120
HPV vaccination date compared to J1 visit	females in Saarland, birth year 1999–2007	KVS	zip code,
		(2013–2019)	birth date (dd/mm/jjjj),
			date J1 (dd/mm/jjjj),
	participation rate birth year 2001–2004		EBM: 01720

### Data analysis and statistical procedures

The data analysis was based on the pseudonymized files in the study database. Descriptive statistics and regression analyses including dependent and independent variables were prespecified. Because this was a full survey rather than a sample considering statutory health insurance billing data, i.e., pseudonymized routine data available from the KVS (2013–2019), IKK (2012–2019) and AOK (2009–2018), we focussed on exploratory statistical analyses.

In the descriptive statistical analysis, absolute and relative frequencies were determined for qualitative data from the parameter list, such as HPV vaccination available, participation in child examinations U8, U9, U11, J1 and the mean was calculated for quantitative variables such as number of doctor visits. The data were additionally stratified by vaccination status: Once regarding vaccination present (yes/no) and the other according to vaccination status complete (yes/no). Logistic regression analyses were conducted, examining the following dependent and independent variables: dependent variable: HPV vaccination available (yes/no); independent variables: participation in U11 and J1 examinations.

Vaccination rates of 15-year-old girls residing in Saarland from 2015 to 2019 were calculated based on the two EBM codes 89110A (first or subsequent vaccination) and 89110B (final vaccination). Considering the age at the start of the vaccination series and the recommended vaccination schedule (9–14-year-old girls: two doses at least five months apart, 15-year-olds: three doses), the completeness of the vaccination series was determined. The completeness of the vaccination series for 9–14-year-old girls at the start of the vaccination series was accordingly given, if a final dose was administered at the latest in the year of the 15th birthday or if two consecutive vaccination doses were administered at an interval of at least five months (two times 89110A). Completion of the vaccination series for 15-year-olds at the start of the vaccination series was given, if a final dose was administered no later than the year of the 15th birthday according to the billing code or if three consecutive vaccination doses were administered (three times 89110A) (see also S1 Table). Analyses and data handling were performed using IBM-SPSS version 25 and R version 4. nQuery Advisor version 7.0 was used for calculations. All confidence intervals are given with a confidence level of 95% and were estimated based on Wald statistics. A two-sided unpaired t-test was applied. Significant differences were accepted if p ≤ 0.05. Data were illustrated with Graph Pad Prism 9 (Graph Pad Software, San Diego, USA).

## Results

First, we determined the frequency of the first HPV vaccination in females in different age groups. For this purpose, we analyzed data from the KVS (years 2014 to 2019). Boys were not included in this analysis because there was no vaccination recommendation for boys at the beginning of the study.

**Fig 1:**
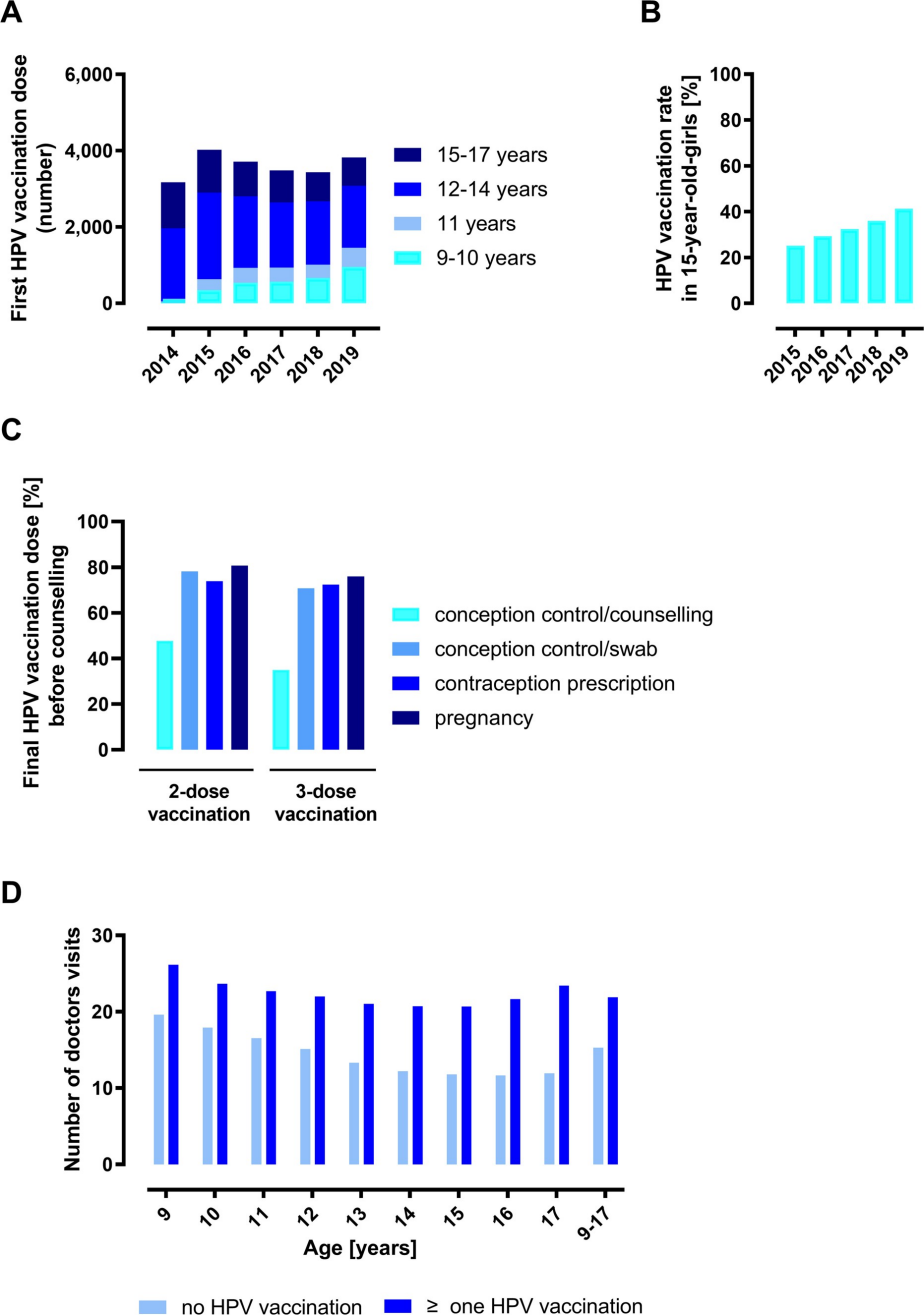
Numbers, rates, and timely finalisation of HPV vaccinations in females. (A) Numbers (absolute frequencies) of the first HPV vaccination by age and years of females in Saarland (KVS data 2014–2019). (B) Rates of fully vaccinated 15-year-old girls in Saarland. Percentage representation of the relative frequencies (data KVS 2015–2019). (C) Timely completion of HPV vaccination. Shown is the proportion of females with EBM code 89110B indicating the final HPV vaccination dose before or after the first date for counselling in the context of conception regulation (EBM code 01821, data KVS 2013–2019), smear collection in the context of conception regulation (EBM code 01825, data KVS 2013–2019), contraceptive prescription (EBM code 01820, data AOK 2009–2018), or pregnancy detection (EBM code 32132, data KVS 2013–2019). Percentage representation of relative frequencies. (D) Average number (absolute frequencies) of physician visits in billing years 2013 to 2019 for female individuals reaching the specified age in 2019 who received no HPV vaccination or at least one HPV vaccination (EBM code 89110A/B, KVS billing data, 2013–2019). Confidence intervals are shown in S9 Table.

After lowering the vaccination age from 12 to 9 years, we observed a clear increase in HPV vaccinations in 2015. The first HPV vaccine dose was increasingly given to younger girls aged 9 to 11 years (increase from 53 to 1,457 from year 2014 to 2019). The first HPV vaccination was more often given at the recommended age of vaccination (9–14 years) than catch-up vaccinations in 2015 to 15-17-year-old females (Fig 1A).

Data from the birth cohorts 2001 to 2004 living in Saarland were used to determine HPV vaccination rates for 2015 to 2019. The rates refer to girls who turned 15 in the corresponding year. For girls who received the first vaccine dose before their 15th birthday, a 2-dose schedule was sufficient for complete vaccination. For completeness, the required vaccine doses had to be administered no later than the end of the year, in which the girls turned 15 years. The interval between two vaccine doses must be at least five months apart, according to the guidelines. For girls who received the first vaccine dose after their 15th birthday, a 3-dose schedule was necessary (see also S1 Table). Our results show that the number of fully HPV-vaccinated 15-year-old girls in Saarland increased steadily between 2015 and 2019. While the HPV vaccination rate was 25.1% in 2015, it was 41.3% in 2019 (Fig 1B).

Subsequently, it was analyzed whether the last HPV vaccination took place before or after a date indicating the start of sexual intercourse, e.g., consultation or smear test in the context of conception regulation, prescription of contraceptives or indication of pregnancy. We would like to emphasize that this evaluation can only refer to those vaccinees for whom the relevant data were available in the analyzed period, i.e., who had undergone the relevant measures or were pregnant. In particular, in the case of prescription for a contraceptive, where data were only available from one health insurance company, or pregnancy, this was only a smaller fraction of those vaccinated (S6 Table). Fig 1C shows that in 47.82% (2-dose vaccination schedule) and 35% (3-dose vaccination schedule) of those vaccinated, the last HPV vaccine dose (as indicated by the EBM code 89110B) was administered before the time of contraceptive counseling, in 78.22% and 70.73%, before the time of contraceptive swab collection, in 73.85% and 72.35%, before the time of prescription for a contraceptive, and in 80.75% and 76.02%, respectively, before the first pregnancy.

We were also interested in the number of physician contacts at the vaccination age and whether there were differences between HPV-vaccinated and non-vaccinated females aged 9–17 years during the 2013–2019 data period. We found that HPV-vaccinated girls had more physician contacts on average than girls who were not vaccinated against HPV. The mean contacts among girls not vaccinated against HPV ranged from 11.67 (in 16-year-old girls) to 19.46 (in 9-year-old girls), whereas the mean contacts among girls vaccinated against HPV ranged from 20.68 (in 15-year-old girls) to 26.18 (in 10-year-old girls) (Fig 1D, 95% confidence interval calculations in S9 Table).

**Fig 2:**
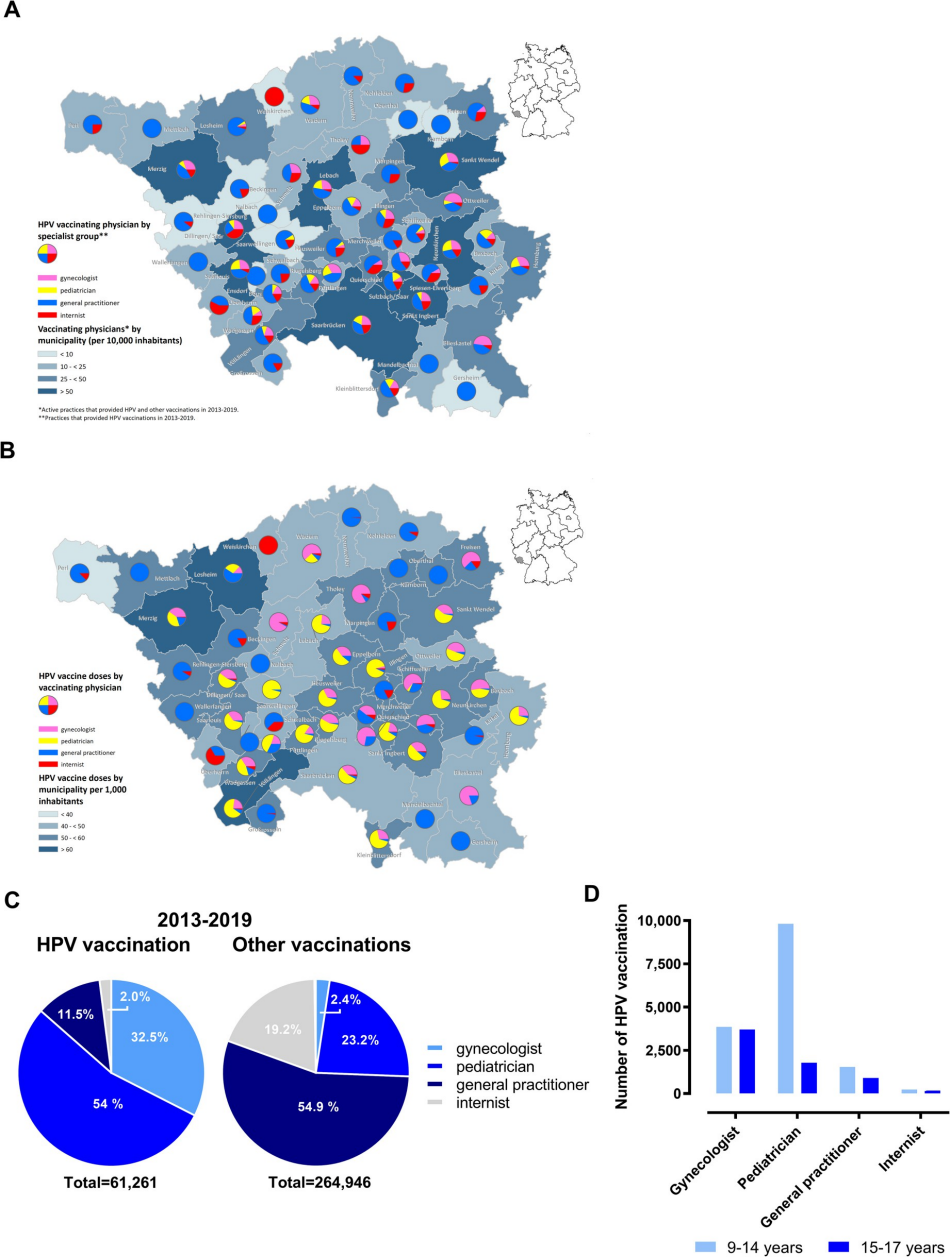
Relationship between HPV vaccination and vaccinating medical specialists. (A) Cartographic distribution of the vaccinating doctors by medical specialty (gynecologists, pink; pediatricians, yellow; general practitioners, blue; internists, red) based on Saarland municipality and per 10,000 inhabitants (data KVS 2013–2019). Administrative boundaries from © GeoBasis-DE / BKG 2020; Data License Germany—Attribution—Version 2.0; Terms of use: http://sg.geodatenzentrum.de/web_public/nutzungsbedingungen.pdf. (B) Cartographic distribution of HPV vaccination doses by medical specialty and by Saarland municipalities, weighted according to the number of inhabitants (data KVS 2013–2019). (C) Administration of HPV vaccinations (total 61,261, left chart) or booster vaccinations (diphtheria, pertussis, tetanus, poliomyelitis, total 264,946, right chart) by different specialist groups. Percentage representation of relative frequencies in pie chart (data KVS 2013–2019). (D) Administration of HPV vaccine doses by medical specialty and age of vaccinees. Presentation of absolute frequencies (data KVS 2013–2019).

To further analyze access to HPV vaccination, we examined the regional distribution of medical specialist groups performing HPV vaccination. In particular, this included gynecologists, pediatricians, general practitioners, and internists, as well as the density of vaccinating doctors per municipality (per 10,000 inhabitants) in Saarland (Fig 2A). There were significant regional differences. In the district of the state capital Saarbrücken and in the vicinity of other larger cities such as St. Wendel, Saarlouis or Neunkirchen) with a higher density of physicians allowed to perform HPV vaccinations, all specialties were represented in a balanced way, whereas in more rural areas gynecologists and pediatricians were clearly less present.

We then evaluated the spatial distribution of medical specialists with the respective administration of vaccine doses. The vaccination rates in the six districts showed little variation (S2 Fig). However, spatial analysis revealed significant regional differences in the administration of HPV vaccine doses per specialist in Saarland municipalities, weighted by population (Fig 2B). In 21 of 52 municipalities, pediatricians were the specialist group that administered the most HPV vaccine doses despite their lower absolute numbers (Fig 2A). In rural communities where pediatrician coverage was lower, HPV vaccine administration was compensated by other specialist groups, especially general practitioners, but also gynecologists.

To obtain a more general picture of vaccine administration in Saarland, we compared the relative contribution of different medical specialties to HPV vaccination and to booster vaccinations against pathogens other than HPV. Between 2013 and 2019, 54% of HPV vaccine doses were administered by pediatricians, 32.5% by gynecologists, 11.5% by general practitioners, and 2% by internists. In contrast, 54.9% of booster vaccinations against diphtheria, pertussis, tetanus, and poliomyelitis were administered by general practitioners and only 23.2% by pediatricians (Fig 2C). It should be noted, however, that the booster vaccinations evaluated in this study were not administered only to children.

Analysis of HPV vaccination by discipline in terms of age of the vaccinees showed that 9- to 14-year-old girls were vaccinated mainly by pediatricians, whereas catch-up HPV-vaccinations of 15- to 17-year-old girls were performed mainly by gynecologists (Fig 2D).

The study also examined how HPV vaccination is delivered in comparison with other preventive measures, such as mandatory childhood check-up examinations U8 (age 46–48 months), U9 (age 60–64 months), U11 (age 9–10 years) or J1 (age 12–14 years). The U11 participation rates could only be evaluated for one health insurance company that provided the data.

**Fig 3:**
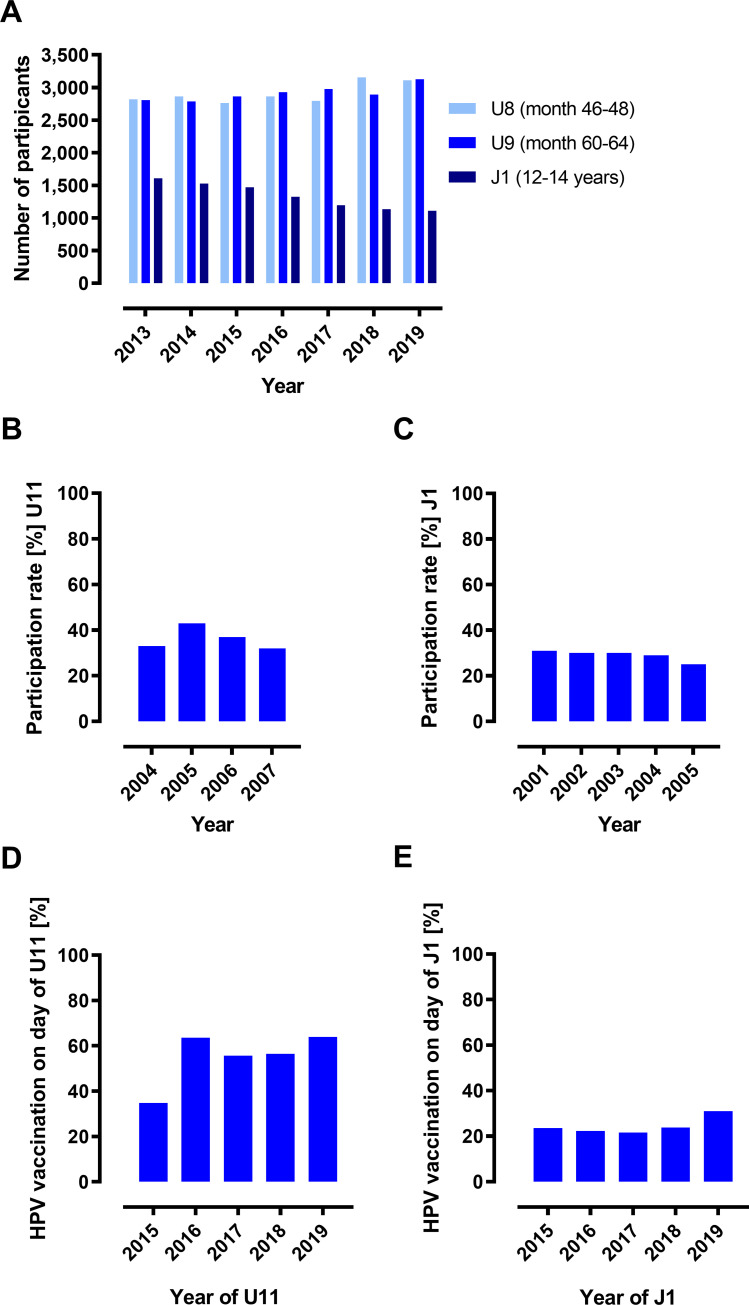
Relationship between HPV vaccination and participation in medical check-ups. (A) Number (absolute frequencies) of preventive medical check-ups among female individuals in Saarland (data KVS 2013–2019). (B) U11 participation rates for girls of birth cohorts 2004–2007. Percentage representation of relative frequencies (obtained from a linked IKK-KVS data set, 2013–2019). (C) J1 Participation rates for female individuals of birth cohorts 2001–2005. Percentage representation of relative frequencies (data KVS 2013–2019). Utilisation of the U11 (D) or J1 (E) appointment for HPV vaccination by females in the year of U11 or J1, respectively. Percentage representation of relative frequencies. Data in (D) were obtained from a linked IKK-KVS data set, 2013–2019; data in (E) from KVS 2015–2019.

Our analysis showed that the numbers of U8 and U9 remained at a consistently high level between 2013 and 2019. In contrast, the number of J1 examinations at the HPV vaccination age of 12–14 years was significantly lower in 2013 and further declined steadily through 2019 (Fig 3A). Participation rates were determined for several birth cohorts in each case and ranged from 32% to 43% for U11 (Fig 3B) and from 25% to 31% for J1 (Fig 3C).

Logistic regression analysis was performed to examine the association between HPV vaccination and U11 and J1 participation during the HPV vaccination age of 9–14 years. Regression analyses for the birth cohorts 2004 and 2005 found that girls who participated in U11 check-up had 5.3 times (95% CI: 4.66–6.11) higher odds of receiving HPV vaccination than girls who did not attend the U11 (p <0.001). Girls who participated in J1 were 10.03 times (95% CI: 8.96–11.22) more likely to receive HPV vaccination than girls who did not attend J1 (p <0.001). In the year of the individual U11 examination, up to 63.9% of those vaccinated were vaccinated against HPV on the day of the U11 examination (Fig 3D). In the year of the individual J1 check-up, up to 31% of those vaccinated against HPV were vaccinated on the day of the J1 examination (Fig 3E). The analysis did, however, not rule out the possibility that girls were vaccinated in an earlier year.

## Discussion

Our study based on routine health insurance data not only provides data on HPV vaccination in a German model region in recent years but also provides new starting points for improving HPV vaccination rates.

We show that HPV vaccination rates in Saarland among 15-year-old girls in 2019 are still well below 50%, far below vaccination rates in other countries such as Australia [27] or in other European countries, e.g., UK [28]. High HPV vaccination success rates in other countries may be based on the early implementation of organized HPV vaccination programs, such as school-based vaccination in United Kingdom, regions of Switzerland, Canada and Australia [29, 30]. In Germany, HPV vaccination is not yet organized but rather opportunistic, although it was called for early after HPV vaccination was introduced [31].

Our data show that overall, HPV vaccination rates have steadily increased since 2015 in Saarland. This was particularly due to an increase in the proportion of 9- to 11-year-old girls among HPV-vaccinated. Lowering of the HPV vaccination to age 9 with the 2-dose schedule [22] resulted in more HPV vaccinations completed. This corresponds to the increase in HPV vaccination rates until 2018 as recently shown in other studies [32, 33], although the exact rates might differ due to differences in the databases (we have limited our evaluations to Saarland resident females) or possibly also calculation modalities.

Prophylactic HPV vaccination should be completed ideally before first sexual intercourse [21] since HPV incidence peaks shortly after sexual debut [34]. Therefore, we examined the timing of completion in those who showed signs of early onset of intercourse. In our study the final HPV vaccination was 35 to 80.75% after the date of a counseling session or first date of smear collection in the context of conception management, the prescription of contraceptives, or pregnancy. This assessment could only refer to those who had undergone the relevant measures or were pregnant. Although the groups studied represent only fractions of those vaccinated, our data suggest that, particularly in these vulnerable subpopulations of females, vaccination was not completed in a timely manner on a substantial scale.

Interestingly, there was no pronounced urban-rural difference in HPV vaccine doses administered in Saarland. In contrast, the specialty of the vaccinating physician played an important role. Pediatricians administered the most HPV vaccine doses and a particularly high proportion of vaccinations to girls of the recommended vaccination age between 9 and 14 years. These results underscored the importance of pediatricians in the successful delivery of HPV vaccination at the recommended age of 9–14 years. Gynecologists, on the other hand, administered more HPV catch-up vaccinations than pediatricians. In contrast, booster vaccinations against other diseases were more likely to be administered by general practitioners. Because the booster vaccinations evaluated in this study were not administered only to children, this may explain the significantly higher booster vaccination rates among general practitioners compared with pediatricians.

In Saarland, in communities where there are fewer pediatricians, general practitioners and gynecologists increasingly administered HPV vaccinations. This suggests that, in addition to pediatricians and gynecologists, general practitioners should be involved in HPV vaccination campaigns, especially in regions with an undersupply of pediatricians, to increase vaccination rates in the future.

In general, the adherence of vaccinated individuals to their physicians appears to play an important role. HPV-vaccinated individuals had significantly more physician contacts during the vaccination age than non-vaccinated individuals. This could indicate that more doctor visits may have increased the likelihood of vaccination. However, HPV-vaccinated individuals might also be more health conscious and therefore visit the doctor more often.

Visiting the pediatrician is especially important to get vaccinated as early as possible. In Germany, children and adolescents who are not yet of vaccination age are invited and reminded to attend the preventive check-ups U3-U9. The pediatricians confirm the attendance and notify the facility issuing the invitation. If a child does not appear even after a reminder, a report is made to the youth welfare office. These early visits are therefore consistently well used. Child and adolescent health check-ups are also offered at the recommended immunization age of 9–14 years (see also S2 Table). In Saarland, there are, however, large differences in the examinations of older children. The J1 examination is conducted nationwide for 12–14-year-olds, but in Saarland without an invitation system. In contrast to the U8 or U9 examinations, participation in the J1 examinations was significantly lower and has continued to decline over the years. The U11 is intended for the younger ones at the age of 9–10 years. However, it is currently not offered and financed by all health insurance funds in Germany, and, even if financed, is not always billed through KV. For U11, the Saarland-wide participation rate could therefore not be determined, because billing data from only one Saarland health insurance company (IKK) were available for evaluation. However, our analysis of the available data showed that there is a significant correlation between the participation in the U11 or J1 check-up examinations and HPV vaccination. Those who attended the corresponding pediatric or adolescent medical examination had a significantly higher chance of being vaccinated against HPV. A positive association has previously been demonstrated by a study on J1 examination and HPV vaccination [35]. Other studies could only show a weak or no correlation with participation in a J1 examination [36].

Our data also show that the day of U11 has a high uptake for HPV vaccination in the year of the U11 examination. In our experience, parents at this age are more likely to accompany their children to the screening appointment than to J1, so parental consent for vaccination can be obtained on the spot and thus vaccination can take place on the same day. The prerequisite for this is that the HPV vaccine is stocked in the doctor’s office, which is the case in Saarland, and should be rolled out nationwide. Organized combined invitation to U11/J1 and HPV vaccination could address issues of different population subgroups and thus reach individuals of different geographical regions, ethnities, socioeconomic status and religions. In addition, HPV vaccination in younger individuals may be more considered as a "normal childhood vaccination" rather than an “STI (sexually transmitted infection) or cancer vaccine” and therefore more disconnected from the issue of sexual activity at a young age.

There have been previous efforts in Germany to increase the HPV vaccination rate through organized programs. A pilot study of school-based HPV vaccination in Germany, which required broad alliances of many public health partners including district administrators, health and education authorities, ministries and associations of physicians, appeared to have reached higher participation rates [37]. A recent detailed analysis of this program by the RKI using billing data from the Association of Statutory Health Insurance Physicians showed that girls living in the district of the school-vaccination program were indeed vaccinated at an earlier age than girls living in adjacent districts [38]. This increased the vaccination rate for 10-year-old girls. However, the vaccination rates for 15-year-old girls did not differ from those of the entire federal state suggesting that there was no net effect on the HPV vaccination rates overall. Notably, within the school-based vaccination program, more girls received their vaccination in a doctor’s office than at school. Even in Austria, a country geographically and culturally close to Germany, the estimated vaccination rate among girls is still below 50% after 20 years of school-based vaccination. Therefore, the success of broad school-based vaccination programs in Germany was recently questioned by the RKI [38].

Our data suggest that the integration of an organized HPV vaccination program into U11 and J1 health check-ups could be very well suited to increase vaccination rates in Germany. This could be an alternative to school-based vaccination, which is apparently difficult to enforce in Germany.

The conclusion made in our study, that implementation of HPV vaccination in U11 and J1 child medical check-ups may increase vaccination rates, is in line with a strong recommendation of the S3 guideline “Vaccination prevention of HPV-associated neoplasms” [39] to improve HPV vaccination rates, for which our study now provides the first data basis. Intervention studies are needed to prove that integrating an organized HPV vaccination program into U11 and J1 health examinations with invitations similar to the children`s screenings at earlier ages can actually increase vaccination rates. According to a resolution of the German “Innovationsausschuss Gemeinsamer Bundesausschuss” this should be taken into account in the further development of future interventions to improve HPV vaccination rates in Germany [40].

### Limitations of the study

Our data are based on statutory health insurance billing data and are therefore more robust than data collected via questionnaires. However, our study also has limitations. (1) The analysis of HPV vaccination on the day of the U11 or J1 examination does not rule out the possibility that girls were vaccinated earlier. (2) U11 billing data were only available from one health insurance company, which might cause a bias in the results. Therefore, the numbers of girls eligible for U11 data were lower than the numbers of girls eligible for other child examinations and the Saarland-wide participation rate could not be determined. (3) 10–15% of people in Germany have private insurance and were therefore not included in our study. Their vaccination behavior may differ from that of those with statutory insurance. (4) We cannot rule out an influence of the socio-economic status or nationality of the children on HPV vaccination rate or attendance of medical check-ups, since such data could not be collected in this study. (5) When analyzing the association between U11 and J1 examinations and vaccination coverage, the "region" of Saarland could not be considered as a potential confounding factor, as the number of cases would have been too small for statistical analysis. (6) Our project started before 2018, when there was no vaccination recommendation for boys in Germany. However, vaccination of boys may provide more robust herd protection. Both aspects, privately insured children including boys, should therefore be considered in a future intervention study to test the effect of organized HPV vaccination.

## Supporting information

S1 Fig Cartographic representation of the cumulative HPV vaccination doses. Billed by the KVS between 2013–2019, administered to females resident in Saarland and neighbouring regions. Administrative boundaries © GeoBasis-DE / BKG 2020; Data License Germany—Attribution—Version 2.0; Terms of use: http://sg.geodatenzentrum.de/web_public/nutzungsbedingungen.pdf.
(TIF)

S2 Fig Proportion of patients from a district in Saarland who have received at least one HPV vaccination (percentage representation of the relative frequencies, data KVS, 2013–2019). (TIF)

S1 Table HPV-vaccination regimens for girls in Germany. (DOCX)

S2 Table Study-relevant child medical check-up system in Germany for children 4 years and older. (DOCX)

S3 Table Number of girls included in the data sets from health insurance funds (AOK, IKK) and the data sets of the association of statutory health insurance physicians Saarland (KVS). (DOCX)

S4 Table Number of girls included in data set for Fig 1A (first HPV vaccination dose). (DOCX)

S5 Table Number of girls included in data set for Fig 1B (HPV vaccination rate). (DOCX)

S6 Table Number of females included in data set for Fig 1C (HPV vaccination before or after an indicated medical indication). Shown are the numbers of females with EBM code 89110B before or after the first date for counselling in the context of conception regulation (EBM code 01821, data KVS 2013–2019), smear collection in the context of conception regulation (EBM code 01825, data KVS 2013–2019), contraceptive prescription (EBM code 01820, data AOK 2009–2018), or pregnancy detection (EBM code 32132, data KVS 2013–2019).
(DOCX)

S7 Table Number of girls included in data set for Fig 1D (doctor visits in 2019). (DOCX)

S8 Table Number of girls included in data sets for Fig 3B and 3C (participating in U11 and J1). (DOCX)

S9 Table 95% Confidence interval calculations Fig 1D. (DOCX)

S1 File Technical procedure for pseudonymization. (DOCX)
