# Supplementary figures and images for: Human papillomavirus vaccination of girls in the German model region Saarland: Insurance data-based analysis and identification of starting points for improving vaccination rates
Source: PLoS One. 2022 Sep 2;17(9):e0273332. doi: 10.1371/journal.pone.0273332 (PMC9439211; doi:10.1371/journal.pone.0273332)

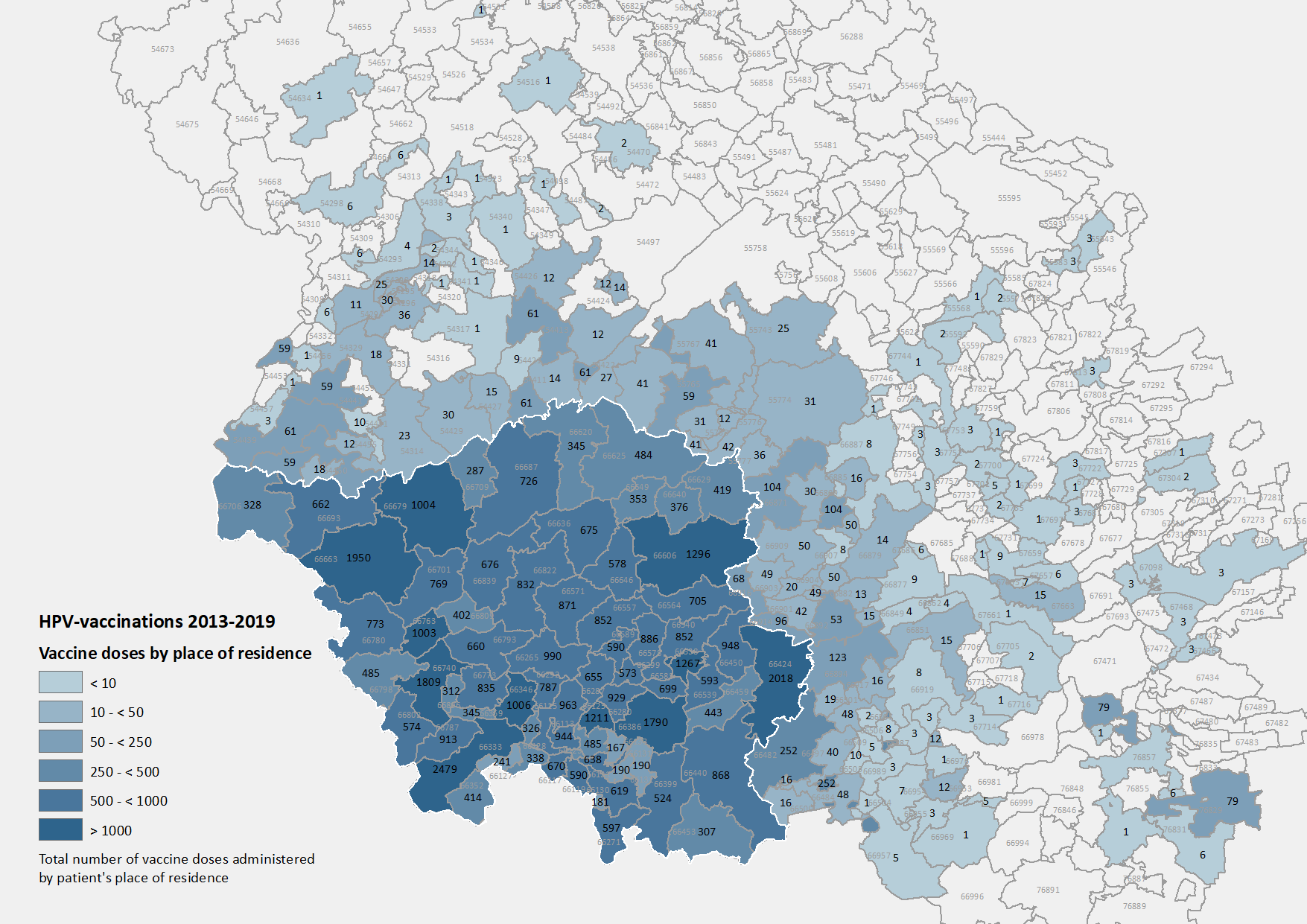

Supplement: S1 Fig — Billed by the KVS between 2013–2019, administered to females resident in Saarland and neighbouring regions. Administrative boundaries © GeoBasis-DE / BKG 2020; Data License Germany—Attribution—Version 2.0; Terms of use: http://sg.geodatenzentrum.de/web_public/nutzungsbedingungen.pdf. (TIF) [file pone.0273332.s001.tif]

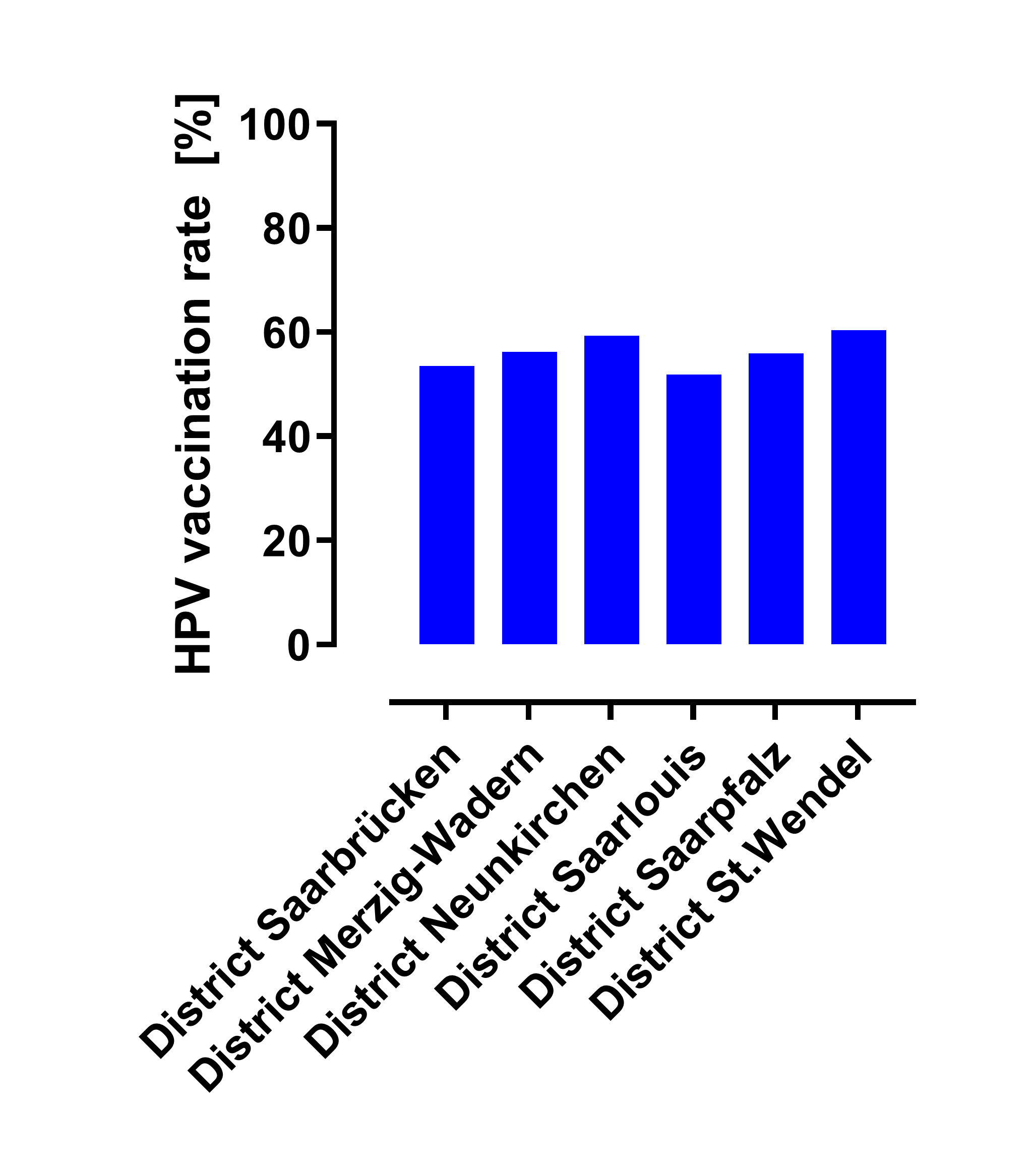

Supplement: S2 Fig — (TIF) [file pone.0273332.s002.tif]
