# Supplementary material for: Human papillomavirus vaccination of girls in the German model region Saarland: Insurance data-based analysis and identification of starting points for improving vaccination rates
Source: PLoS One. 2022 Sep 2;17(9):e0273332. doi: 10.1371/journal.pone.0273332 (PMC9439211; doi:10.1371/journal.pone.0273332)
Supplement: S1 Table — (DOCX) [file pone.0273332.s003.docx]

**S1 Table. HPV vaccination regimens for girls in Germany**

| **Girls`s Age** | **12-17 years** | **9-14 years** | **> 15 years** |
| --- | --- | --- | --- |
| **Time period** | Until 2014 | Since 2014* | Since 2014* |
| **Number of doses** | 3-dose | 2-dose | 3-dose |
| **Vaccination schedule**  **Cervarix® (month)** | Cervarix®: 0, 1, 6 | 0, 6 month (<5 month interval: 3 doses) | 0, 1, 6 |
| **Vaccination schedule**  **Gardasil® (month)** | Gardasil ®: 0, 2, 6 | 0, 6 month (<5 month interval: 3 doses) | 0, 2, 6 |

*Decision for implementation in November 2014
