# Supplementary material for: Human papillomavirus vaccination of girls in the German model region Saarland: Insurance data-based analysis and identification of starting points for improving vaccination rates
Source: PLoS One. 2022 Sep 2;17(9):e0273332. doi: 10.1371/journal.pone.0273332 (PMC9439211; doi:10.1371/journal.pone.0273332)
Supplement: S2 Table — (DOCX) [file pone.0273332.s004.docx]

**S2 Table. Study-relevant child medical check-up system in Germany for children 4 years and older** (check-ups below 4 years are not mentioned here)

| **Check-up** | **Age** | **Special features** |
| --- | --- | --- |
| U8, U9 | 4 and 5 years | Mandatory, financed by all health insurances, invitation and reminder system for parents |
| U10 | 7-8 years | Voluntary, financed by most health insurances |
| U11 | 9-10 years | Voluntary, financed by most health insurances |
| J1 | 12-14 years | Voluntary, financed by all health insurances, invitation letter for parents in some parts of Germany (not in Saarland) |
