# Supplementary material for: Human papillomavirus vaccination of girls in the German model region Saarland: Insurance data-based analysis and identification of starting points for improving vaccination rates
Source: PLoS One. 2022 Sep 2;17(9):e0273332. doi: 10.1371/journal.pone.0273332 (PMC9439211; doi:10.1371/journal.pone.0273332)
Supplement: S3 Table — (DOCX) [file pone.0273332.s005.docx]

**S3 Table. Number of girls included in the data sets from health insurance funds (AOK, IKK) and the data sets of the association of statutory health insurance physicians Saarland (KVS)**

|  | **Number of females insured** | **Number of females insured,** Saarland: zip codes 66110 to 66840 | **Number of females living in Saarland** |
| --- | --- | --- | --- |
|  | | | |
| **AOK (2009-2018)** | 221,812 | 151,147 | 511,088 (2009-2018, mean) |
| **IKK (2012 as of 3rd quarter-2018)** | 101,782 | 81,513 | 507,185 (2012-2018, mean) |
| **KVS (2013-2019)** | 892,815 | 603,841 | 506,004 (2013-2019, mean) |
| Data sets with linked records | | | |
| **IKK-KVS (2013-2019)** | 80,452 | 65,157 | 506,593 (2013-2019, mean) |

During the recording period, persons were added to the data record over the years. Persons once recorded remained in the data set, even if no further billing data were listed due to departures from the Saarland or death. In addition, inflows into Saarland and births have increased the size of the data set over time.
