# Supplementary material for: Human papillomavirus vaccination of girls in the German model region Saarland: Insurance data-based analysis and identification of starting points for improving vaccination rates
Source: PLoS One. 2022 Sep 2;17(9):e0273332. doi: 10.1371/journal.pone.0273332 (PMC9439211; doi:10.1371/journal.pone.0273332)
Supplement: S4 Table — (DOCX) [file pone.0273332.s006.docx]

**S4 Table. Number of girls included in data set for Fig 1A (First HPV vaccination dose)**

| **Year\Age** | **9-10 years** | **11 years** | **12-14 years** | **15-17 years** |
| --- | --- | --- | --- | --- |
| 2014 | 22 | 31 | 1,917 | 1,203 |
| 2015 | 346 | 288 | 2,267 | 1,122 |
| 2016 | 544 | 388 | 1,872 | 912 |
| 2017 | 565 | 371 | 1,707 | 838 |
| 2018 | 663 | 349 | 1,665 | 755 |
| 2019 | 956 | 501 | 1,631 | 734 |
