# Supplementary material for: Human papillomavirus vaccination of girls in the German model region Saarland: Insurance data-based analysis and identification of starting points for improving vaccination rates
Source: PLoS One. 2022 Sep 2;17(9):e0273332. doi: 10.1371/journal.pone.0273332 (PMC9439211; doi:10.1371/journal.pone.0273332)
Supplement: S5 Table — (DOCX) [file pone.0273332.s007.docx]

**S5 Table**. **Number of girls included in data set for Fig 1B (HPV vaccination rate)**

| **Year** | **Number of girls** |
| --- | --- |
| **2015** | 1,296 |
| **2016** | 1,403 |
| **2017** | 1,481 |
| **2018** | 1,536 |
