# Supplementary material for: Human papillomavirus vaccination of girls in the German model region Saarland: Insurance data-based analysis and identification of starting points for improving vaccination rates
Source: PLoS One. 2022 Sep 2;17(9):e0273332. doi: 10.1371/journal.pone.0273332 (PMC9439211; doi:10.1371/journal.pone.0273332)
Supplement: S6 Table — Shown are the numbers of females with EBM code 89110B before or after the first date for counselling in the context of conception regulation (EBM code 01821, data KVS 2013–2019), smear collection in the context of conception regulation (EBM code 01825, data KVS 2013–2019), contraceptive prescription (EBM code 01820, data AOK 2009–2018), or pregnancy detection (EBM code 32132, data KVS 2013–2019). (DOCX) [file pone.0273332.s008.docx]

**S6 Table. Number of females included in data set for Fig 1C (HPV vaccination before or after an indicated medical indication)**

|  |  | **Medical indication for doctors` visits** | | | |
| --- | --- | --- | --- | --- | --- |
|  |  | Conception control/counselling | Conception control/swab | Contraception prescription | Pregnancy |
| **2-dose vaccination** | **Before final HPV vaccination** | 2,172 | 1,681 | 257 | 541 |
|  | **After final HPV vaccination** | 2,370 | 468 | 91 | 129 |
| **3-dose vaccination** | **Before final HPV vaccination** | 2,287 | 2,938 | 806 | 929 |
|  | **After final HPV vaccination** | 4,247 | 1,216 | 308 | 293 |
