# Supplementary material for: Human papillomavirus vaccination of girls in the German model region Saarland: Insurance data-based analysis and identification of starting points for improving vaccination rates
Source: PLoS One. 2022 Sep 2;17(9):e0273332. doi: 10.1371/journal.pone.0273332 (PMC9439211; doi:10.1371/journal.pone.0273332)
Supplement: S7 Table — (DOCX) [file pone.0273332.s009.docx]

**S7 Table.** **Number of girls included in data set for Fig 1D (Doctor visits in 2019)**

| **Age** | **No HPV vaccination** | **One or more HPV vaccination** |
| --- | --- | --- |
| **9** | 3,830 | 194 |
| **10** | 3,290 | 622 |
| **11** | 3,037 | 894 |
| **12** | 2,579 | 1,247 |
| **13** | 2,154 | 1,557 |
| **14** | 1,920 | 1,972 |
| **15** | 1,789 | 2,206 |
| **16** | 1,785 | 2,198 |
| **17** | 1,853 | 2,458 |
| **9-17** | 22,237 | 13,348 |
