# Supplementary material for: Human papillomavirus vaccination of girls in the German model region Saarland: Insurance data-based analysis and identification of starting points for improving vaccination rates
Source: PLoS One. 2022 Sep 2;17(9):e0273332. doi: 10.1371/journal.pone.0273332 (PMC9439211; doi:10.1371/journal.pone.0273332)
Supplement: S8 Table — (DOCX) [file pone.0273332.s010.docx]

**S8 Table.** **Number of girls included in data sets for Figs 3B and C (Participating in U11 and J1**)

| **Birth year** | **Number of girls U11** | **Number of girls J1** |
| --- | --- | --- |
| **2001** | **-** | 1,518 |
| **2002** | **-** | 1,379 |
| **2003** | **-** | 1,284 |
| **2004** | 751 | 1,250 |
| **2005** | 725 | 1,056 |
| **2006** | 701 | **-** |
| **2007** | 795 | **-** |
