# Supplementary material for: Human papillomavirus vaccination of girls in the German model region Saarland: Insurance data-based analysis and identification of starting points for improving vaccination rates
Source: PLoS One. 2022 Sep 2;17(9):e0273332. doi: 10.1371/journal.pone.0273332 (PMC9439211; doi:10.1371/journal.pone.0273332)
Supplement: S9 Table — (DOCX) [file pone.0273332.s011.docx]

**S9 Table. 95% Confidence interval calculations Figure 1D.**

|  | **Mean number of doctors visits [95% CI]** |
| --- | --- |
| **9 year olds** |  |
| **No HPV Vaccination** | 19.64 [19.24; 20.05] |
| **≥ 1 HPV Vaccination** | 26.18 [24.54; 27.81] |
| **10 year olds** |  |
| **No HPV Vaccination** | 17.95 [17.52; 18.38] |
| **≥ 1 HPV Vaccination** | 23.65 [22.88; 24.43] |
| **11 year olds** |  |
| **No HPV Vaccination** | 16.54 [16.11; 16.96] |
| **≥ 1 HPV Vaccination** | 22.68 [22.01; 23.36] |
| **12 year olds** |  |
| **No HPV Vaccination** | 15.12 [14.67; 15.57] |
| **≥ 1 HPV Vaccination** | 22.00 [21.43; 22.57] |
| **13 year olds** |  |
| **No HPV Vaccination;** | 13.33 [12.87; 13.79] |
| **≥ 1 HPV Vaccination** | 21.03 [20.51; 21.55] |
| **14 year olds** |  |
| **No HPV Vaccination** | 12.22 [11.75; 12.69] |
| **≥ 1 HPV Vaccination** | 20.74 [20.29; 21.19] |
| **15 year olds** |  |
| **No HPV Vaccination** | 11.80 [11.31; 12.29] |
| **≥ 1 HPV Vaccination** | 20.68 [20.24; 21.12] |
| **16 year olds** |  |
| **No HPV Vaccination** | 11.67 [11.18; 12.16] |
| **≥ 1 HPV Vaccination** | 21.66 [21.20; 22.12] |
| **17 year olds** |  |
| **No HPV Vaccination** | 11.94 [11.40; 12.48] |
| **≥ 1 HPV Vaccination** | 23.45 [22.97; 23.92] |
| **9-17 year olds** |  |
| **No HPV Vaccination** | 15.28 [15.12; 15.44] |
| **≥ 1 HPV Vaccination** | 21.88 [21.69; 22.06] |
