# Supplementary material for: Human papillomavirus vaccination of girls in the German model region Saarland: Insurance data-based analysis and identification of starting points for improving vaccination rates
Source: PLoS One. 2022 Sep 2;17(9):e0273332. doi: 10.1371/journal.pone.0273332 (PMC9439211; doi:10.1371/journal.pone.0273332)
Supplement: S1 File — (DOCX) [file pone.0273332.s012.docx]

## Technical procedure for pseudonymization

For all personal information, instead of transmitting identity data in plain text (last name, last name part 2, last name part 3 or additions, first name, first name part 2, first name part 3, maiden name, maiden name part 2, maiden name part 3, former name, former name part 2, Earlier Name Part 3, Date of Birth, Phonetically Standardized Last Name, Phonetically Standardized First Name, Phonetically Standardized Birth Name, Phonetically Standardized Earlier Name, Title, Title Part 2) only the corresponding hashed encrypted tokens derived were provided. Ciphering was performed using standardized plaintext features (one-way hash and symmetric IDEA encryption) by the KVS. This precluded recovery of the plaintext data. The linkage of the data was done using a record linkage procedure, which is routinely used in cancer registration and was adapted for the purposes of this study by the Saarland Cancer Registry [1].

**References**

1. Hoffmeister M, Holleczek B, Zwink N, Stock C, Stegmaier C, Brenner H. Screening for Bowel Cancer: Increasing Participation via Personal Invitation. Deutsches Arzteblatt international. 2017;114(6):87-93.
